# Lessons from making the Structural Classification of Proteins (SCOP) and their implications for protein structure modelling

**DOI:** 10.1042/BST20160053

**Published:** 2016-06-15

**Authors:** Antonina Andreeva

**Affiliations:** * MRC Laboratory of Molecular Biology, Francis Crick Avenue, Cambridge CB2 0QH, U.K.

**Keywords:** homology modelling, metamorphic proteins, protein structure evolution, Structural Classification of Proteins (SCOP)

## Abstract

The Structural Classification of Proteins (SCOP) database has facilitated the development of many tools and algorithms and it has been successfully used in protein structure prediction and large-scale genome annotations. During the development of SCOP, numerous exceptions were found to topological rules, along with complex evolutionary scenarios and peculiarities in proteins including the ability to fold into alternative structures. This article reviews cases of structural variations observed for individual proteins and among groups of homologues, knowledge of which is essential for protein structure modelling.

## Introduction

Over the past two decades, the Structural Classification of Proteins (SCOP) database has become an essential resource in many areas of protein research [1]. Initially designed to assist structural biologists in the analysis of structural similarities between proteins, SCOP facilitated the development of tools and algorithms and it has been successfully used in protein structure prediction and large-scale genome annotations [2,3]. SCOP also contributed to our understanding of protein repertoire, including how proteins relate to each other and how their structures and functions evolved [4]. Each grouping in the classification was the product of a careful, systematic analysis of protein structures and a detailed knowledge of protein function and evolution. Many distant evolutionary relationships between proteins were first discovered during their analysis for classification in SCOP [5–7]. Some of these have never been described in the literature and thus the SCOP database has become a repository for many interesting research findings.

The notion of protein evolution, incorporated in SCOP, allowed grouping of proteins based not only on their structural features but also on their common evolutionary origin. Depending on the degree of evolutionary divergence and structural similarity, discrete units (*domains*) are hierarchically organized into *families* and *superfamilies*. These are further grouped into structural *folds*, defined by the domains’ topology and architecture, and *classes* reflecting their secondary structure composition. The classification of proteins in SCOP depends on their relationships to proteins with known 3D structure and their identification typically includes a sequence similarity search against a database of structurally characterized proteins. Close evolutionary relationships between proteins, e.g. *family* relationships, are usually detectable with sequence search methods such as BLASTP or FASTA. At the *superfamily* level, most of the distant relationships are detectable using iterative PSI-BLAST, hidden Markov models or profile–profile searches [8]. These preliminary classification steps are very similar to the initial steps for the identification of templates in template-based protein structure modelling (also known as homology or comparative modelling) [9–12], which usually begin by searching a sequence database of proteins with known 3D structures using the target sequence as a query. Once a suitable template is selected, all current methods create an alignment of the target and template sequences and this alignment is further used as input to build a 3D model for the target protein. Template-based methods rely on two important assumptions: that proteins fold into one stable folded structure and that homologous proteins fold into similar structures. Current methods can produce reliable and accurate protein structure models when suitable templates are selected and the degree of structural conservation between the full length target and template protein is substantial.

Since the SCOP database was established in 1995, the amount of structural data has grown nearly 40-fold. The classification protocol has changed over this time, allowing better evaluation of sequence–structure relationships for classified proteins and the quality of alignments produced by different sequence comparison algorithms [13]. Numerous exceptions observed to topological rules, along with complex evolutionary scenarios and unusual protein features prompted the development of SCOP2, a successor of the SCOP database [14]. Here, I review selected cases of structural variations and peculiarities in individual proteins and among group of homologues. Knowledge of these cases may be of use in essential steps of protein structure modelling such as the selection of structurally and biologically relevant templates or for improving the target-template sequence alignments by considering evolutionary information about the structural variations of both the target and template proteins.

## Conformational transitions in proteins

**Figure 1:**
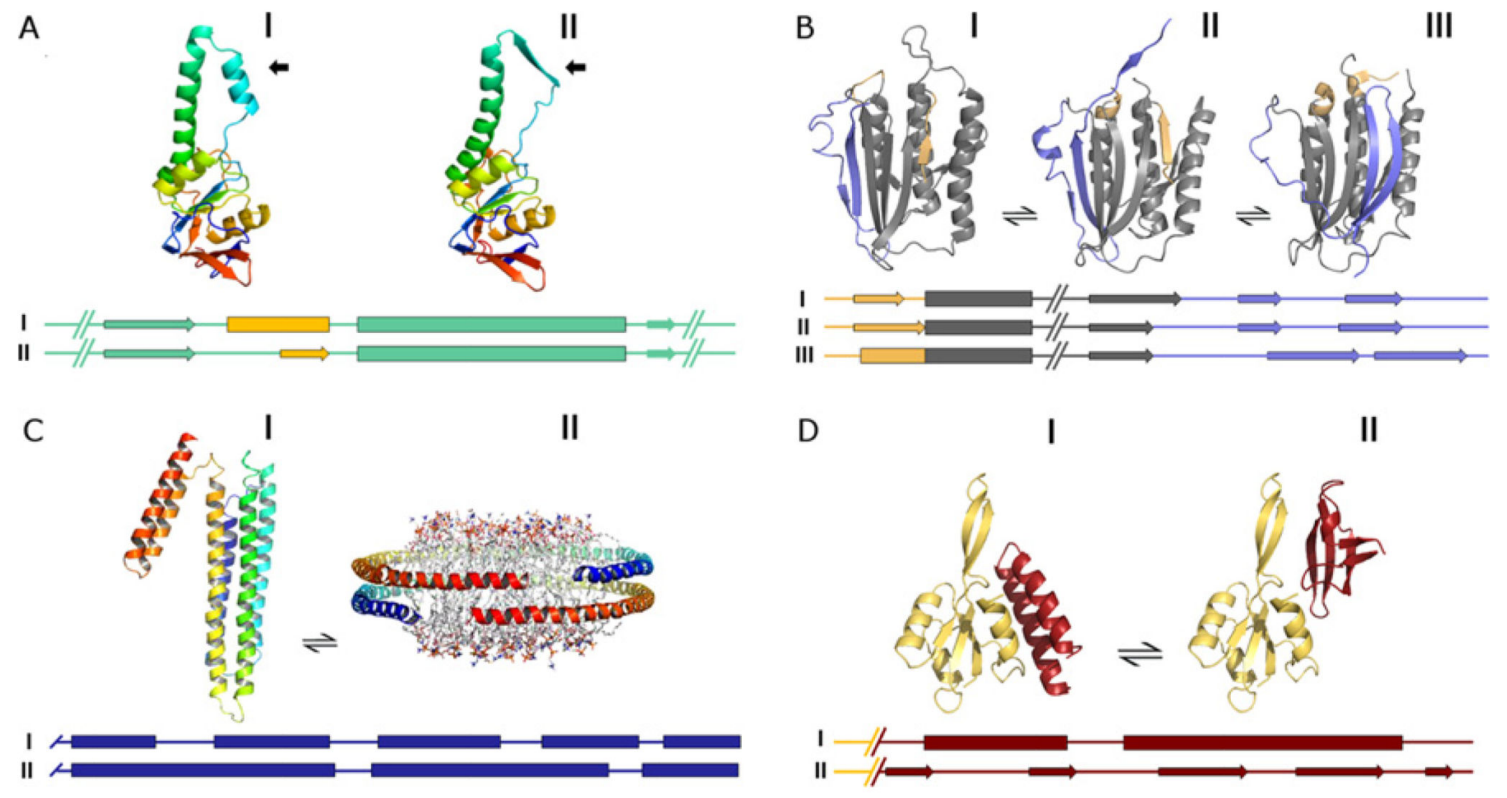
Conformational transitions in proteins Side by side comparison of alternative conformers of: (**A**) *α*-apical domain of the thermosome: I) isolated domain (PDB 1ASS), II) domain from the closed thermosome (PDB 1A6E); the region that undergoes a secondary structural transition from *α* to *β* is indicated with a black arrow and coloured in orange in the secondary structure plot; (**B**) Mad2: I) O-Mad2 (PDB 1DUJ), II) I-Mad2 (PDB 3GMH, chain B), III) C-Mad2 (PDB 3GMH, chain E); the regions that undergo a structural change and a *β*-to-*α* transition are coloured in light blue and in orange respectively; (**C**) apolipoprotein A: I) lipid-free form (PDB 2A01), II) lipid-bound form (PDB 2MSD); (**D**) RfaH: I) closed form (PDB 2OUG), II) open form (PDB 2LCL). The secondary structure plots refer only to portions of each structure that are shown in particular colours (e.g. green in (**A**), grey in (**B**), blue in (**C**), red in (**D**)). All figures were prepared using Pymol (http://www.pymol.org).

Conformational changes in proteins have been known for a long time and are crucial to many biological processes [15]. These range from a subtle side-chain displacement or a loop flexibility to a large domain motion involving hinge regions that are not constrained by packing forces. In some proteins, short ‘chameleon sequences’ can undergo more dramatic changes and adopt alternative secondary structures. Chameleon sequences are more common to intrinsically disordered proteins [16], but they can also be found in globular domains. For example, in hypoxia-inducible factor prolyl hydroxylase 2 (PHD2), a region located in the active site vicinity (*β*2*β*3 loop) undergoes transition from an extended *β* to an irregular conformation upon binding to HIF*α* peptide (PDB 3HQR) [17]. Similarly, upon oligomer formation, an *α* to *β* conformational transition is observed in the *α*-apical domain of the thermosome (Figure 1A) [18]. These conformational changes, although quite dramatic, usually involve relatively short stretches of amino acid residues.

Some proteins, however, undergo much larger structural rearrangements, leading to a conformational transition from one stable folded state to another. These so-called metamorphic proteins [19] exist in multiple conformations and undergo conformational transitions that involve a major rearrangement of both their secondary structural elements and their entire hydrogen bonding network, repacking of their interior and, in most known cases, exposure of a new binding interface. This new binding interface is usually associated with a new function that is exhibited by one of the conformers but not by the others, and hence the structural transitions observed in metamorphic proteins play an important role in their molecular function. One of the first known examples are the serpins, which upon proteolytic cleavage undergo irreversible structural changes associated with their inhibitory mechanism [20]. Their close homologue ovalbumin (30% identity), for example, is not subject to similar conformational changes [21]. More recently, several proteins have been shown to exist in an equilibrium of multiple conformational states and can reversibly change their structures. Mitotic arrest deficient 2 (Mad2) was first described to exist in three conformations, latent (open) O-Mad2, (intermediate) I-Mad2 and activated (closed) C-Mad2 [22,23]. In the latter, the C-terminal region refolds into an irregular structure, the so-called ‘safety belt’, and a *β*-hairpin that replaces the N-terminal strand in the O-Mad2 structure. The N-terminal strand shifts and undergoes a transition from *β* to *α* conformation in the activated conformer. The formation of the ‘safety belt’ is used to topologically entrap Mad2 binding partners containing the so-called MIM motif. In complex with its binding partners, C-Mad2 can recruit additional copies of O-Mad2 and convert them into an intermediate I-Mad2 that is a structural hybrid of the two conformers (Figure 1B). In contrast with Mad2, lymphotactin undergoes complete rearrangement of all stabilizing interactions in order to convert from a monomeric chemokine fold to a dimeric *β*-sandwich fold [24]. The chemokine-like conformer binds to XCR1 GPCRs whereas the dimeric conformer lacks this ability, but instead it interacts with cell-surface glycosaminoglycans.

In many aspects chloride intracellular channel protein 1 (CLIC1) has the most complex scenario for structure and function transitions. CLIC1 is a chloride ion channel that exists as both a globular soluble and a transmembrane form. Soluble CLIC1 exists in equilibrium between monomeric and dimeric states. The monomeric form has a typical GST fold with N-terminal thioredoxin-like domain that undergoes a structural transition to an all *α*-helical conformation upon dimerization [25]. This conformational switch results in the exposure of a large hydrophobic surface that contributes to the dimeric interface. Only the dimeric form can interact with membrane lipids. Upon binding to the lipid surface, the same N-terminal region becomes a transmembrane helix that penetrates the lipid bilayer and via self-association forms the channel pore [26].

Conformational transitions induced by a change of the environment are intrinsic features of some *α*-helical proteins. For example, upon contact with lipids apolipoprotein A undergoes a change from a four helical up-and-down bundle to a ring-like structure that wraps around the lipids (Figure 1C) [27]. The lipid-free form of apolipoprotein is involved in various interactions with cellular receptors whereas the lipid-bound form is involved in a lipid transport. Similarly, saposins undergo conformational changes from closed monomeric to open dimeric form in the presence of lipids [28,29]. The death domain of protein kinase Pelle (Pelle-DD) adopts a six helical bundle in solution, characteristic of the death domain family, but in the presence of MPD (2-methyl-2,4-pentanediol), the structure of Pelle-DD refolds into a single helix [30].

A striking structural and functional transition is observed for the RfaH transcription factor, the C-terminal domain of which undergoes a transition from an *α*-helical hairpin to an SH3 *β*-barrel, converting it from a transcription into a translation factor (Figure 1D) [31]. RfaH is a member of a conserved ubiquitous multigene family of transcription factors. The *α*-helical conformer masks the RNA polymerase binding interface in the N-terminal domain and this autoinhibition is essential to avoid functional interference with its paralogue, NusG. Both RfaH conformers are functionally active: the *α*-hairpin binds to the ribosome and activates translation whereas the *β*-barrel form has a function similar to NusG.

Many more examples of structural transitions are known, such as for fibronectin [32], T-cell receptor *α* [33], KaiB [34], etc. Little is known about the exact mechanisms that drive these conformational changes. The functional requirement for some proteins to form and maintain an accurate and specific active or binding site probably exerts a strong selective pressure to adopt only one stable folded structure. For other proteins, however, conformational transitions provide an elegant way of switching between different molecular functions. Our current state of knowledge about the large structural rearrangements of certain proteins does not have any predictive power but it has some important implications for protein structure modelling. Particularly, it is essential for the selection of relevant templates and in finding the structural conformer that is more suitable for modelling. Given that many methods use non-redundant sequence databases derived by using sequence similarity clustering, it is currently up to the user to identify the most appropriate template and its relevant conformer for a particular modelling problem.

## Conservation of protein structure during evolution

**Figure 2:**
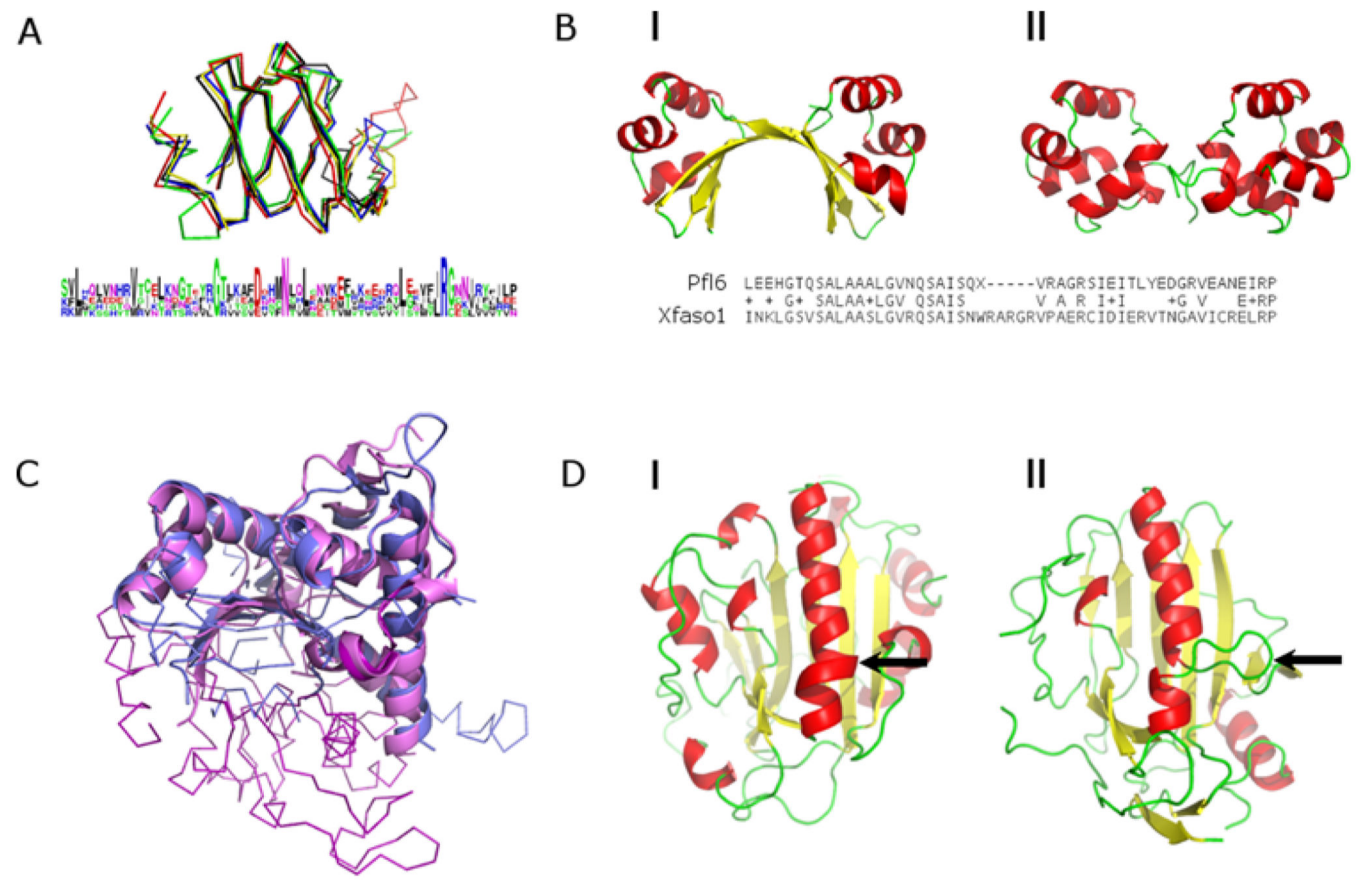
Evolution of protein structures (**A**) Superposition of Sm-proteins. Structures are shown in ribbon and coloured as follows: in yellow – Sm D1 (PDB 1B34, chain A), in green – Sm D2 (PDB 4PJO, chain D), in blue – Sm D3 (PDB 1D3B, chain A), in red – Sm B (PDB 1D3B, chain B), in black – Sm F (PDB 1N9R, chain A). A sequence logo showing the degree of amino acid conservation derived from the structure-based sequence alignment is shown below. (**B**) Side by side comparison of the structures of two Cro-proteins. I) Pfl6 (PDB 2PIJ) and II) Xfaso1 (PDB 3BD1); BLASTP pair-wise sequence alignment with 45% identity over 55 residues and one 5 residue gap; (**C**) fold decay event in the glutamate synthase central domain; the FMN-binding domain is shown in purple (PDB 1OFD, chain A, residues 840–1210) and the central domain in blue (PDB 1OFD, chain A, residues 490–735); structurally equivalent regions are shown in cartoon and the rest in ribbon. (**D**) Large insertion in an *α*-helix in the structures of two nonspecific endonucleases. I) Nuclease A from *Anabaena sp.* (PDB 1ZM8); II) Nuclease A from *Streptococcus agalactiae* (PDB 4QH0).

Proteins are the evolutionary products of various molecular events operating at gene level such as point mutations, nonhomologous recombination, transposition, juxtaposition, exon rearrangement, gene or exon duplications, etc. Mutations of many amino acids in proteins do not affect or have only marginal effect on structure and stability. Therefore, unless there is a selective pressure for a conformational change, the structures of homologous proteins should be similar. Generally, proteins performing the same molecular function diverge with speciation of organisms and hence their structures tend to be more conserved than their sequences. An example is the structural conservation observed in the SCOP family of Sm-like proteins. These proteins fold into a partly open *β*-barrel and associate in hetero- or homoheptameric ring structures [35] that serve as platforms for versatile protein–protein and protein–RNA interactions. The requirement to maintain the oligomer symmetry that is essential for the protein function exerts a strong evolutionary pressure to maintain the 3D shape and, despite the low sequence similarity (10–30% sequence identity over 65 residues), all members have very similar structures (Figure 2A). The most conserved sequence features of this family are two Gly residues that play a role in maintaining the barrel curvature typical for all Sm-like proteins. At the level of ∼50% sequence identity, it is likely that proteins have very similar 3D structures. There are, however, exceptions to this rule and there are homologous proteins having very similar sequences but globally different structures. In the Cro family of repressors, for instance, Pfl6 and Xfaso1, share 45% sequence identity over 55 residues. Their structures retain the local structural similarity of the DNA binding motif at their N-termini but, despite a high sequence similarity, they adopt very different structures at their C-termini [36]. In Xfaso1 this region is *α*-helical whereas in Pfl6 it folds into *β*-sheet stabilized by dimerization (Figure 2B).

Events such as transposition, nonhomologous recombination, alternative splicing etc., can result in insertions or deletions and sometimes can significantly alter the structure of protein gene products. For example, the proteins belonging to the SCOP *α*/*β* hydrolase superfamily exhibit large deletions or insertions of secondary structural elements and even entire domains in order to accommodate different substrates. The common structural core of these homologous proteins, however, remains conserved, particularly near the active site and the nucleophile elbow motif (PDB 5AJH, 4J7A, 1THG, 3I2K). The evolutionary scenario with the glutamate synthase family is quite different: the FMN-binding domain was duplicated and fused and then the duplicated domain underwent a large deletion of three *β*/*α* units, resulting in an incomplete barrel (Figure 2C). Deletion events of this kind that affect the structural cores of homologous proteins are not uncommon. A similar event occurred in the structure of a nonfluorescent flavoprotein in which the remaining structural parts retain significant sequence similarity (36% identity) to its homologue, luciferase (PDB 1NFP, 1LUC). Insertions and deletions can also occur within secondary structural elements. Some members of the nonspecific endonucleases superfamily, for instance, contain a loop bisecting a long *α*-helix that borders the enzyme active site (Figure 2D). The length of this loop varies between 9 and 13 residues in different homologues but interestingly the conformation of the *α*-helix before and after the insertion does not deviate.

Other scenarios of protein structure evolution and structural changes in homologous protein families have been described elsewhere [37–42]. The knowledge of protein families, their conserved features and structural variations is a prerequisite for better quality model building. Human expertise is also essential to distant homology recognition and the modelling of homologous but structurally divergent proteins. Looking back in retrospect, two approaches in protein structure prediction, distant homology recognition in CASP2 (Critical Assessment of protein Structure Prediction) and hybrid template assemblies in CASP4, were pioneered by the main author of SCOP, Alexey Murzin. His detailed knowledge of protein structures allowed his successful prediction in CASP4 of a novel topology for target T0104, which still remains unique among the known P-loop containing proteins [43].

Using evolutionary information about the target and the template can be helpful to improve the quality of the target/template alignments or to define specific alignment constraints in template-based modelling. Evolutionary information, however, can sometimes introduce a bias and affect the performance of some secondary structure prediction methods. This can happen in multigene families where a particular structural feature has been lost in some lineages. For instance, secondary structure prediction methods that exploit evolutionary information fail to predict the second helix in the p53 tetramerization domain in bony fishes that is otherwise absent from other vertebrate p53 proteins [44].

## Proteins with unusual topologies

**Figure 3:**
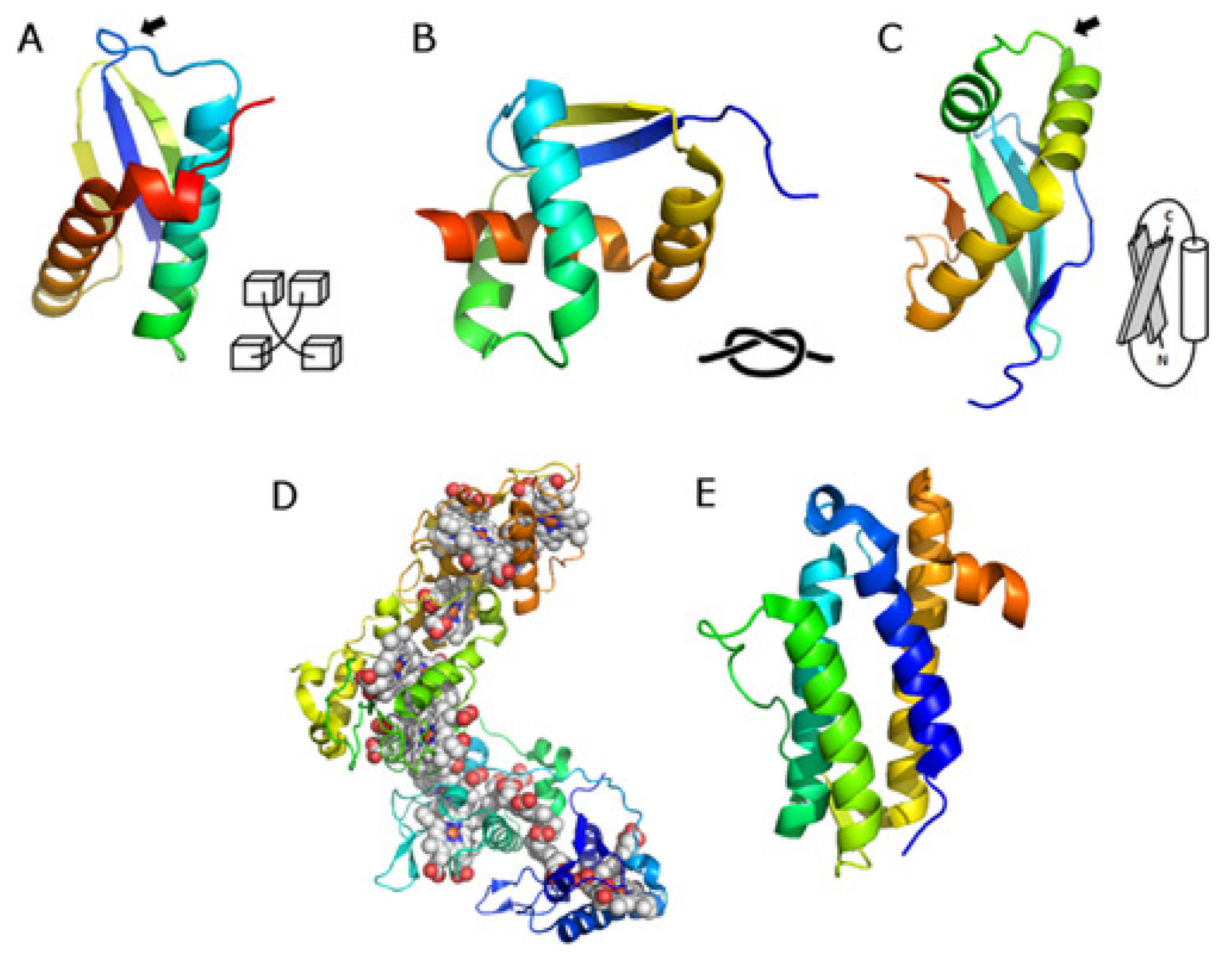
Examples of proteins with unusual topologies (**A**) Loop crossing in the structure of DinI (PDB 1GHH); (**B**) trefoil knot in the MJ0366 structure (PDB 2EFV); (**C**) left-handed *β*–*α*–*β* connection in the structure of a protein with unknown function shew_3726 (PDB 2GPI); a black arrow indicates the location of the unusual topological feature; a schematic drawing of each feature is shown next to each structure for clarity, (**D**) structure of the hexadeca-haem cytochrome Hmc that does not possess a compact hydrophobic core (PDB 1GWS); (**E**) high contact order structure of DinB protein (PDB 2F22).

Folding pathways of proteins tend to follow an energetically favourable route leading to a stable, low energy conformation. Several empirical rules were established during early analyses of protein structures, underlining basic topological principles and preferences [45–47]. Some of these postulated that secondary structural elements that are adjacent in sequence make a contact in three dimensions, that is, protein structures tend to have a low contact order [48]. In order to fold into a stable globular structure, it was reasoned that *α*-helical and *β*-sheet secondary structure elements should associate tightly and pack closely to form a hydrophobic core of a protein. Topological features such as crossing loops and left-handed *β*–*α*–*β* connections were considered energetically unfavourable and very rare. Similarly, knots in the polypeptide chain were postulated as highly improbable due to a large entropic barrier to folding and the intrinsically difficult process of formation of knotted topology. Nowadays, exceptions to each of these rules have been observed (Figure 3). Some of these previously considered rare and improbable features appear to be characteristic of highly represented protein families. The superfamily of RNA methyltransferases containing a deep trefoil knot, for example, consists of numerous families, many members of which have been structurally characterized recently [49]. Another example is the vast expanding superfamily of DinB/YfiT-like putative metalloenzymes that fold into high contact order structures and probably originated from an interlocked dimeric homologue (Figure 3E). Prediction of long range interactions in proteins still remains a difficult problem. Topological restraints in structure modelling are now increasingly being used in order to improve the prediction accuracy. Their stringency should be carefully considered or complex folds and knotted topologies may never be predicted.

## Concluding remarks

This review was an attempt to provide a brief, and very selective, overview of our current understanding of how proteins evolved and function, and give a hint of possible implications to structure modelling. It is noteworthy that although exceptions have been found for nearly every rule defined in the past, these do not disprove the rule. Many homologous proteins fold into similar structures and their structures are more conserved than their sequences. Importantly, every group of related proteins has its own evolutionary history and perhaps underwent events that may not be observed in other protein families. Evolutionary changes are not restricted to the peripheral elements of a protein domain but can also affect the structural core. Many proteins adopt a single, unique, well defined three-dimensional structure under native conditions. By contrast, others exist in multiple conformational states and hence provide new insights into how protein structures and functions can evolve through the process of conformational transitions.

## Abbreviations

CASP: critical assessment of protein structure prediction
CLIC1: chloride intracellular channel protein 1
Mad2: mitotic arrest deficient 2
SCOP: Structural Classification of Proteins
